# A novel method for investigating *Burkholderia cenocepacia* infections in patients with cystic fibrosis and other chronic diseases of the airways

**DOI:** 10.1186/s12866-016-0811-7

**Published:** 2016-09-01

**Authors:** Christiaan D. M. Wijers, Ryan Vagedes, Christine Weingart

**Affiliations:** Department of Biological Sciences, Denison University, 100 West College Street, Granville, OH 43023 USA

**Keywords:** *Burkholderia*, *cenocepacia*, Tissue culture, CF sputum, PCD sputum, Model

## Abstract

**Background:**

*Burkholderia cenocepacia* is a Gram-negative, opportunistic pathogen that is a cause of morbidity and mortality in patients with cystic fibrosis (CF). Research efforts over the past few decades contributed to our understanding of these infections by identifying virulence factors. However, little is known about how this pathogen adapts to the harsh environment found inside the CF airways, which is characterized by a unique mucus containing high concentrations of inflammatory markers. The current study developed a novel model to further investigate this phenomenon.

**Results:**

Monolayers of human A549 lung carcinoma cells (HLCCs) were exposed to a mixture of artificial CF sputum medium (ASMDM) in tissue culture growth medium, and subsequently infected with *B. cenocepacia* K56-2 for 24 h. The data showed that this model supported *B. cenocepacia* growth. In addition, consistent with similar studies using current models such as CF airway tissue samples, HLCC viability was reduced by more than 70 % when grown in 60 % ASMDM and infected with *B. cenocepacia* compared to mock-infected controls and medium alone. Furthermore, the amount of *B. cenocepacia* cells associated with the HLCC monolayer was more than 10 times greater in 60 % ASMDM when compared to medium controls.

**Conclusions:**

These findings suggest that HLCC monolayers in 60 % ASMDM serve as a valid alternative to study *B. cenocepacia* infections in patients with CF, and possibly other chronic diseases of the airways. Furthermore, the results obtained in this study suggest an important role for CF sputum in *B. cenocepacia* pathogenesis.

**Electronic supplementary material:**

The online version of this article (doi:10.1186/s12866-016-0811-7) contains supplementary material, which is available to authorized users.

## Background

Mucus plays an important protective role in our host defenses by trapping inhaled particles such as pathogens that are subsequently transported out of the airways by ciliated epithelial cells. For this mucociliary clearance to work effectively, the cilia must constantly be moving in the thin, moist layer of mucus. Some diseases such as cystic fibrosis (CF) and primary ciliary dyskinesia (PCD) impair this clearance mechanism resulting in chronic lung infections. More specifically, mutations in the cystic fibrosis conductance regulator cause a disruption in the transfer of Cl- across the cell membrane. Consequently, the mucus layer becomes thick and dehydrated preventing the successful removal of the particles. PCD patients have defective cilia structure causing the cilia to beat abnormally. Previous research indicated that there exists striking similarities in terms of mucus biophysical and chemical properties between PCD and CF sputum [1–3]. In addition to impaired mucociliary clearance, both diseases have a neutrophil-dominated inflammation in the airways. These conditions are challenging for many bacteria, however highly adaptable pathogens such as *Pseudomonas aeruginosa*, *Staphylococcus aureus*, *Haemophilus influenza* survive in the CF and PCD airways (see review [2, 4]) and *B. cenocepacia* thrives in the CF airways (see review [4–6]).

*Burkholderia cenocepacia* is a member of the *Burkholderia cepacia* Complex (BCC) that comprises 18 species that are Gram-negative opportunistic pathogens [7]. It is innately resistant to a wide array of antibiotics including aminoglycosides, quinolones, and β-lactams [8], (see review [4]). It possesses a variety of virulence factors such as cable pili, biofilm formation, degradative enzymes (see review [9]), (see review [10]), and it is transmitted from person-to-person [11] and from the environment [12]. While the majority of CF patients infected with *B. cenocepacia* experiences a gradual deterioration of lung function [6, 8], nearly 20 % of infected CF patients experience “cepacia syndrome”. This is a fatal case of necrotizing pneumonia sometimes associated with septicemia that may lead to death within one year [6, 13]. Because of these attributes, this pathogen poses a serious threat to CF patients.

Over the past decade progress has led to a greater understanding of how *B. cenocepacia* causes disease in CF patients (see review [9]). However, because there is still no definite cure, further research is needed, on how this pathogen adapts to the harsh CF airways as such information could lead to new forms of treatment. Unfortunately, for investigative purposes, it can be very difficult to acquire sputum or tissue samples from CF patients, and there exists a need for an alternative, readily available method to investigate *B. cenocepacia* infections in CF patients and patients with similar chronic diseases of the airways.

Fung et al. [14] developed an artificial sputum medium (ASMDM) that approximates the sputum found in CF patients in terms of components, concentrations of the components, and physical properties. In their study, they found that *Pseudomonas aeruginosa*, another opportunistic pathogen commonly found in CF airways (see review [4, 15]) grew normally and deeply invaded the ASMDM, suggesting that ASMDM mimicked the CF lower airway mucus well [14]. In addition, the fact that ASMDM seems to be a good substitute for CF mucus and a good growth medium for *P. aeruginosa* suggests that ASMDM would be a suitable growth medium for *B. cenocepacia*.

Nevertheless, using ASMDM by itself is not sufficient to simulate the in vivo conditions in the lungs of CF patients, as this model lacks live host cells that can respond to a *B. cenocepacia* infection. Saijan et al. investigated *B. cenocepacia* infections in the presence of well-differentiated human CF cells and in non-CF human lung epithelial cells [16]. They found that infected CF cells contained significantly more bacteria in both the mucosal layer and inside the cell layer when compared to infected non-CF cells [16]. This study showed that *B. cenocepacia* penetrated the mucosal and cellular layers which is comparable to its activity in the CF airways.

Our study investigated the accuracy and effectiveness of ASMDM on a monolayer of human A549 lung carcinoma cells, as a model to investigate *B. cenocepacia* infections in CF and PCD patients. Although the literature indicates that *B. cenocepacia* has never been isolated from PCD airways, similarities in mucus biophysical and chemical properties and bacterial flora between the sputa seem striking enough to suspect that *B. cenocepacia* may be able to colonize PCD airways as well. Therefore, this model could be a valid alternative to investigate *B. cenocepacia* infections in CF patients, and it might be used to investigate *B. cenocepacia* or other infections in PCD patients as well.

## Methods

### Cell line and propagation

Human A549 lung carcinoma cells (HLCCs) (American Type Culture Collection, Manassas, VA) were maintained in 1 mL aliquots at −80 °C. Prior to experimental use, 1 mL HLCCs was thawed in a 37 °C water bath, transferred to a Falcon T25 vial (Fisher Scientific, Hanover Park, IL) containing 5 mL Ham’s F-12 complete medium (American Type Culture Collection), and incubated at 37 °C (7 % CO_2_). At confluency, HLCCs were detached from the flask surface with 2 mL 1X trypsin-EDTA (Mediatech, Inc., Manassas VA), and added to 10 mL Ham’s F-12 complete medium in a Falcon T75 vial (Fisher Scientific, Hanover Park, IL).

### Bacterial strain and culture conditions

*Burkholderia cenocepacia* K56-2 [17] was grown in 10 mL Luria-Bertani broth. Cultures were incubated for 24 h at 37 °C with shaking (150 rpm).

### Preparation of artificial sputum medium (ASMDM)

ASMDM was prepared in 30 mL aliquots as described by Fung et al. [14]. Porcine stomach mucin (Sigma-Aldrich, St. Louis, MO), DNA from salmon testes (Sigma-Aldrich, St. Louis, MO), potassium chloride (Fisher Scientific, Hanover Park, IL), sodium chloride (Fisher Scientific, Hanover Park, IL), diethylene triamine pentaacetic acid (Fluka Analytical, St. Louis, MO), casamino acids (Acros, Hanover Park, IL), and bovine serum albumin (Sigma-Aldrich, St. Louis, MO) were dissolved in 25 mL sterile dH_2_O (final concentrations: 10 mg/mL, 1.4 mg/mL, 2.2 mg/mL, 5 mg/mL, 5.9 mg/mL, 5 mg/mL, and 10 mg/mL, respectively). Egg yolk emulsion (0.15 mL) (Becton Dickinson) and the antibiotics ampicillin (1 ug/mL; (Fisher Scientific, Hanover Park, IL) and penicillin (1 ug/mL; Sigma-Aldrich, St. Louis, MO) were added to revent contamination. Tetracycline was omitted because *B. cenocepacia* is sensitive to this antibiotic [18]. The suspension was stirred for 5 min at room temperature to dissolve the DNA and mucin. The pH was adjusted to 6.5, and sterile dH_2_O was added to 30 mL ASMDM was stored at 4 °C, but warmed to 37 °C before addition to cells.

### Determination of optimal concentration of ASMDM for HLCCs and *B. cenocepacia*

HLCCs (0.5 mL) were added to gas-permeable 24-well plates (Coy Laboratory Products, Inc., Grass Lake, MI) at 4x10^5^ cells/mL. The plates were incubated at 37 °C (7 % CO_2_) for 24 h to form a monolayer. The medium was discarded, cells were washed once with 0.5 mL 1X HBSS (Mediatech, Manassas, VA), and then exposed to either 0.5 mL Ham’s F-12 complete medium, ASMDM suspended in Ham’s F-12 complete medium at varying concentrations (20 %, 40 %, 60 %, or 80 %), or 100 % ASMDM. Two wells were not manipulated (i.e. not washed, and medium not replaced), and were designated as the T_24_ control. Plates were then incubated at 37 °C (7 % CO_2_). After 24 h, the supernatant was removed from all wells (including T_24_ wells), washed with 0.5 mL 1X HBSS, and then washed with 0.5 mL 1X trypsin-EDTA. Cells were detached from the well surface with 0.5 mL 1X trypsin-EDTA at 37 °C (7 % CO_2_) for 10 min. The cell density (cells/mL) was determined. The cell viability (% live cells) was measured with Trypan blue staining.

To determine the optimal concentration of ASMDM for *B. cenocepacia* viability, 1 mL of the overnight culture was centrifuged at 14,000 rpm for 2 min, washed in 1 mL saline, and then suspended in 1 mL saline. This suspension (10 μL) was added to non-gas permeable 24-well plates (Fisher Scientific, Hanover Park, IL) containing either 0.5 mL Ham’s F-12 complete medium, ASMDM suspended in Ham’s F-12 complete medium at varying concentrations (20 %, 40 %, 60 %, or 80 %), or 100 % ASMDM. The plate was incubated at 37 °C (7 % CO_2_). After 24 h, CFU/mL per well was determined via viable cell plating. The CFU/mL of the saline suspension was determined via viable cell plating; this is the T_0_ control.

### Assessment of the effects of *B. cenocepacia* on HLCCs with 60 % ASMDM

HLCCs suspended in Ham’s F-12 complete medium (0.5 ml) was added to a gas-permeable 24-well plate at a density of 3 × 10^5^ cells/mL and incubated at 37 °C (7 % CO_2_) for approximately 24 h until confluent. The medium was discarded. Cells were washed once with 0.5 mL 1X HBSS, and then exposed to 0.5 mL medium or 60 % ASMDM in Ham’s F-12 complete medium. Wells without HLCCs were filled with 0.5 mL medium or 100 % ASMDM. Wells were either treated with 10 μL of *B. cenocepacia* in saline at MOI 0.3-5 or mock-infected with saline. Two wells were not manipulated for the duration of the experiment to assess the cell density (T_24_ controls). The plates were incubated at 37 °C (7 % CO_2_). After 24 h, damage to the monolayer was assessed with an inverted Zeiss axiovert 40 CFL microscope (W. Nuhsbaum, Inc., McHenry, IL), a microscope-mounted camera (AmScope, Irvine, CA), and Leica LAS v4.6.2. software. Damage was defined as the presence of gaps in the monolayer. To assess the cell density and viability of HLCCs, half the wells were treated as follows. The remaining supernatant was discarded, cells were rinsed twice with 0.5 mL 1X HBSS, and once with 0.5 mL 1X trypsin-EDTA. Wells were then filled with another 0.5 mL 1X trypsin-EDTA and stored at 37 °C (7 % CO_2_) for 10 min. The cell density and viability were determined.

The remaining wells were used to measure bacterial density. To quantify the bacteria in the supernatant, 0.2 ml was plated for cell viability. To quantify internal and adherent bacteria, the wells were treated as follows. The remaining supernatant was removed, cells were washed twice with 0.5 mL 1X HBSS, and once with 0.5 mL sterile dH_2_O. Wells were then filled with 1 mL sterile dH_2_O and incubated at 37 °C (7 % CO_2_) for one hour to degrade the monolayer and lyse the HLCCs. Samples (0.2 mL) were removed for viable cell plating.

## Results

### ASMDM concentration for optimal HLCC viability

**Fig. 1:**
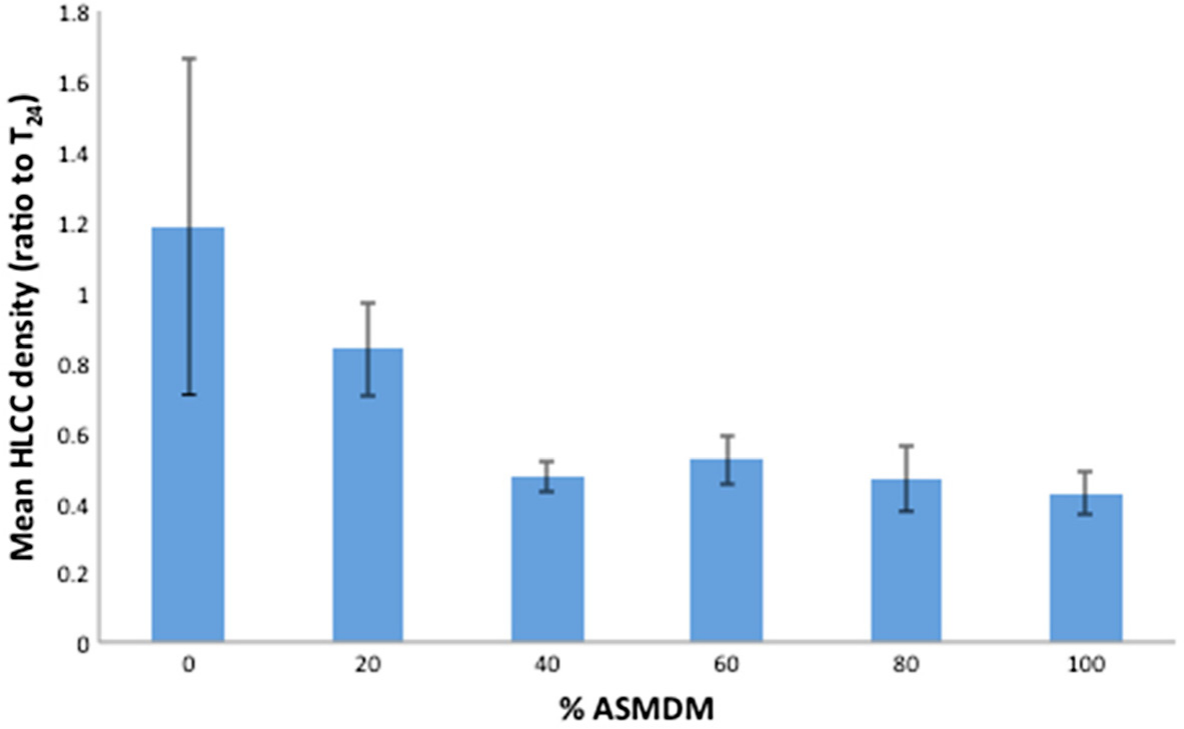
Effect of ASMDM on the density of human lung carcinoma cells A549 (HLCCs). Confluent HLCCs grown in gas permeable 24-well plates were exposed to varying concentrations of ASMDM in Ham’s F-12 complete medium. After 24 h, the cell density (cells/mL) of HLCCs was compared to the cell density of T_24_ control HLCCs. T_24_ control consisted of HLCC monolayers in Ham’s F-12 complete medium that were not manipulated (i.e. not washed, and medium was not replaced) for the duration of the experiment. There are no statistically significant differences between any of the different combinations (oneway ANOVA, *p* = 0.07). Values are averages from four independent experiments each with duplicate wells. Bars show standard deviation

**Fig. 2:**
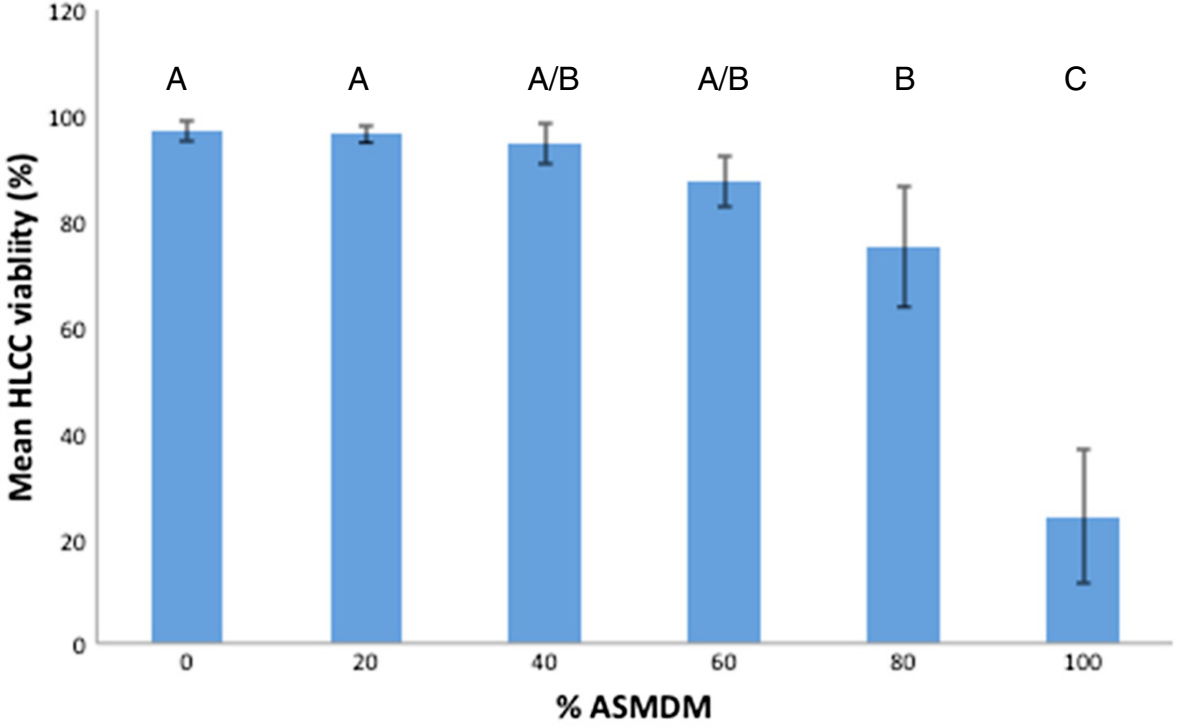
The effect of ASMDM on the viability of HLCCs. Confluent HLCCs grown in gas permeable 24-well plates were exposed to varying concentrations of ASMDM in Ham’s F-12 complete medium. After 24 h, mean viability was determined using Trypan blue staining. There are statistically significant differences in mean cell viability between the different combinations (ANOVA, *p* < 0.0001). A Tukey-HSD post-hoc test indicated that the mean cell viability in 100 % ASMDM is statistically different from the 0, 20, 40, 60, or 80 % ASMDM (*p* < 0.0001 for all), and that the mean cell viability in 80 % ASMDM is statistically different than the 0 or 20 % ASMDM (*p* = 0.0336 and *p* = 0.0404, respectively). Values are averages of four independent experiments with duplicate or triplicate wells. Bars show standard deviation. Variables with different letters are statistically different (oneway ANOVA, *p* < 0.05)

We used ASMDM to simulate the CF mucous composition and viscosity [14], gas-permeable plates to allow for appropriate gas exchange, and human A549 lung carcinoma cells (HLCC) to proxy for CF epithelial cells because studies showed that *Burkholderia* invades this cell line [19, 20]. First, we determined the concentration of ASMDM necessary to maintain HLCC density. Our results showed that the average cell density ratio in 0 % ASMDM was greater compared to the other concentrations. However, the differences were not significantly different (oneway ANOVA, *p* = 0.07) (Fig. 1) indicating that the cell density was not influenced by the ASMDM concentration. Next, we measured the HLCC viability. When the cells were exposed to 0 through 60 % ASMDM the cell viability was high (around 90 %), but decreased to 75 % in 80 % ASMDM and 24 % in 100 % ASMDM (Fig. 2). The differences in cell viability between the different concentrations of ASMDM were significantly different (oneway ANOVA, *p* < 0.0001). A Tukey-HSD post-hoc test indicated that the mean cell viability in 100 % ASMDM is significantly different compared to 0 through 80 % ASMDM (*p* < 0.0001 for all), and that the mean cell viability in 80 % ASMDM is significantly different compared to 0 or 20 % ASMDM (*p* = 0.0336 and *p* = 0.0404, respectively).

### The effect of ASMDM on the viability of *B. cenocepacia*

**Fig. 3:**
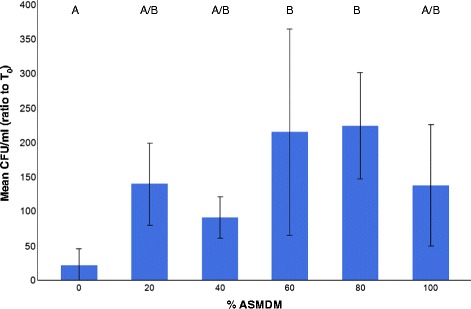
The effect of ASMDM on *B. cenocepacia* growth. *B. cenocepacia* was added to non-gas permeable 24-well plates containing ASMDM diluted in Ham’s F-12 complete medium and incubated at 37 °C (7 % CO_2_). After 24 h, mean CFU/mL of *B. cenocepacia* in each well was compared to the starting culture of *B. cenocepacia* (T_0_). There are statistically significant differences in mean CFU/mL ratio between 0 and 60–80 % (oneway ANOVA, *p* = 0.03). Values are averages of four independent experiments with duplicate or triplicate wells. Bars show standard deviation. Variables with different letters are statistically different (oneway ANOVA, *p* < 0.05)

To determine whether ASMDM affected *B. cenocepacia* growth, 10 μL of bacterial suspension (approximately 5 × 10^8^ CFU/mL) was added to non-gas-permeable plates containing ASMDM. *B. cenocepacia* grew in all concentrations of ASMDM, but grew better (>100-fold) in wells containing at least 20 % ASMDM than in Ham’s F-12 complete medium alone. A Tukey-HSD post hoc test showed that 0 % is significantly different from the 60 and 80 % ASMDM (oneway ANOVA, *p* = 0.03) (Fig. 3). These results suggested that *B. cenocepacia* grew well in at least 20 % ASMDM (Fig. 3). Therefore, in combination with the HLCC viability and cell density data, we used 60 % ASMDM for our model.

### The effect of *B. cenocepacia* on HLCCs in 60 % ASMDM

**Fig. 4:**
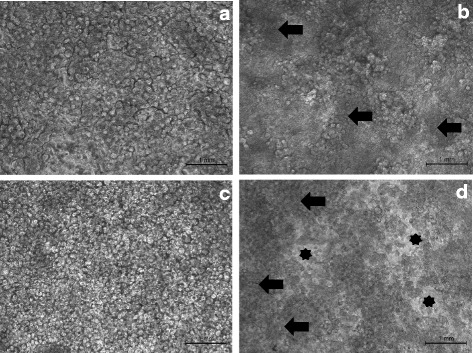
The effect of *B. cenocepacia* infection on the integrity of HLCC monolayers. Confluent HLCCs were subjected to Ham’s F-12 complete medium (**a** and **b**) or 60 % ASMDM (**c** and **d**), infected with *B. cenocepacia* at MOI 0.3-5 (**b** and **d**) or mock-infected with saline (**a** and **c**), and incubated at 37 °C (7 % CO_2_). After 24 h, HLCC monolayers were assessed for damage with an inverted Zeiss axiovert 40 CFL microscope (1000X magnification). HLCC monolayers in both mock-infected wells are confluent with no damage (**a** and **c**). HLCC monolayer in Ham’s F-12 complete medium with *B. cenocepacia* shows no sign of damage, although a dense mass is floating in the well. Because this is seen in infected wells only, we think the aggregations are clouds of bacterial cells floating in the supernatant (*arrows*) (**b**). HLCC monolayer in 60 % ASMDM with *B. cenocepacia* shows damage as indicated by gaps in the monolayer (*black stars*). Clouds of bacterial cells can be seen as well (*arrows*) (**d**)

We measured the effect of infection by first examining the integrity of the monolayer. Mock-infected HLCC monolayers in Ham’s F-12 complete medium or 60 % ASMDM showed no signs of monolayer damage (Fig. 4a and c). The infected HLCC monolayer in Ham’s F-12 complete medium showed no signs of damage either, although clouds of bacterial cells were seen floating in the supernatant (Fig. 4b). However, the HLCC monolayer in 60 % ASMDM infected with *B. cenocepacia*, showed signs of damage as indicated by gaps in the HLCC monolayer. Clouds of bacterial cells were observed as well (Fig. 4d). An additional image file shows this in more detail [see Additional file 1]. We think these clouds are dense masses of bacteria because they are seen only in the infected wells. These observations indicated that *B. cenocepacia* caused a breach in the integrity of the established monolayer in the 60 % ASMDM.

**Fig. 5:**
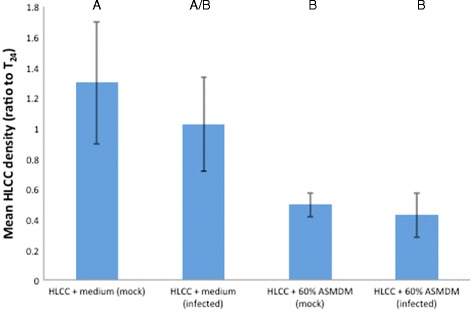
The effect of *B. cenocepacia* on HLCC density in 60 % ASMDM. Confluent HLCCs were exposed to 60 % ASMDM diluted in Ham’s F-12 complete medium, infected with *B. cenocepacia* at MOI 0.3-5 or mock-infected with saline, and incubated at 37 °C (7 % CO_2_). After 24 h, the cell density (cells/mL) of each well was compared to the cell density of T_24_ control HLCCs. T_24_ control consisted of HLCCs in Ham’s F-12 complete medium that were not manipulated (i.e. not washed, and medium not replaced) for the duration of the experiment. There are statistically significant differences in mean cell density ratio between the different treatments (ANOVA, *p* = 0.0037). A Tukey-HSD post-hoc test indicated that the mean cell density ratio in infected HLCCs + 60 % ASMDM is significantly different than in mock-infected HLCCs + Ham’s F-12 complete medium (*p* = 0.0055). The mean cell density ratio in mock-infected HLCCs + 60 % ASMDM is significantly different than in mock-infected HLCCs + Ham’s F-12 complete medium (*p* = 0.0135). Values are averages of three independent experiments with single wells. Bars show standard deviation. Variables with different letters are statistically different (oneway ANOVA, *p* < 0.05)

**Fig. 6:**
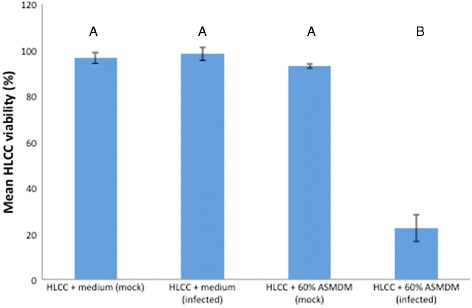
The effect of *B. cenocepacia* on HLCC viability in 60 % ASMDM. Confluent HLCCs were exposed to 60 % ASMDM diluted in Ham’s F-12 complete medium, infected with *B. cenocepacia* at MOI 5 or mock-infected with saline, and incubated at 37 °C (7 % CO_2_). After 24 h, HLCC viability was determined using Trypan blue staining. There are statistically significant differences in mean cell viability between the different treatments (ANOVA, *p* < 0.0001). A Tukey-HSD post-hoc test indicated that the mean cell viability of infected HLCCs + 60 % ASMDM is significantly different than mock-infected HLCCs + Ham’s F-12 complete medium, infected HLCCs + Ham’s F-12 complete medium, and mock-infected HLCCs + 60 % ASMDM (*p* < 0.0001 for all). Values are averages of three independent experiments with single wells. Bars show standard deviation. Variables with different letters are statistically different (oneway ANOVA, *p* < 0.05)

We also examined the mean cell density and viability. The mean cell density ratio significantly decreased in mock-infected and infected HLCCs in 60 % ASMDM compared to mock-infected HLCCs in Ham’s F-12 complete medium (Tukey-HSD post hoc, *p* = 0.0135 and *p* = 0.0055, respectively) (Fig. 5). There was no significant difference compared to the infected HLCCs in Ham’s F-12 complete medium. However, the cell viability dropped significantly in infected HLCCs grown in 60 % ASMDM (Tukey-HSD post hoc, *p* < 0.0001) (Fig. 6). These results indicated that 60 % ASMDM negatively affected the cell density (Figs. 1 and 5), but the bacterial infection negatively affected the cell viability.

**Fig. 7:**
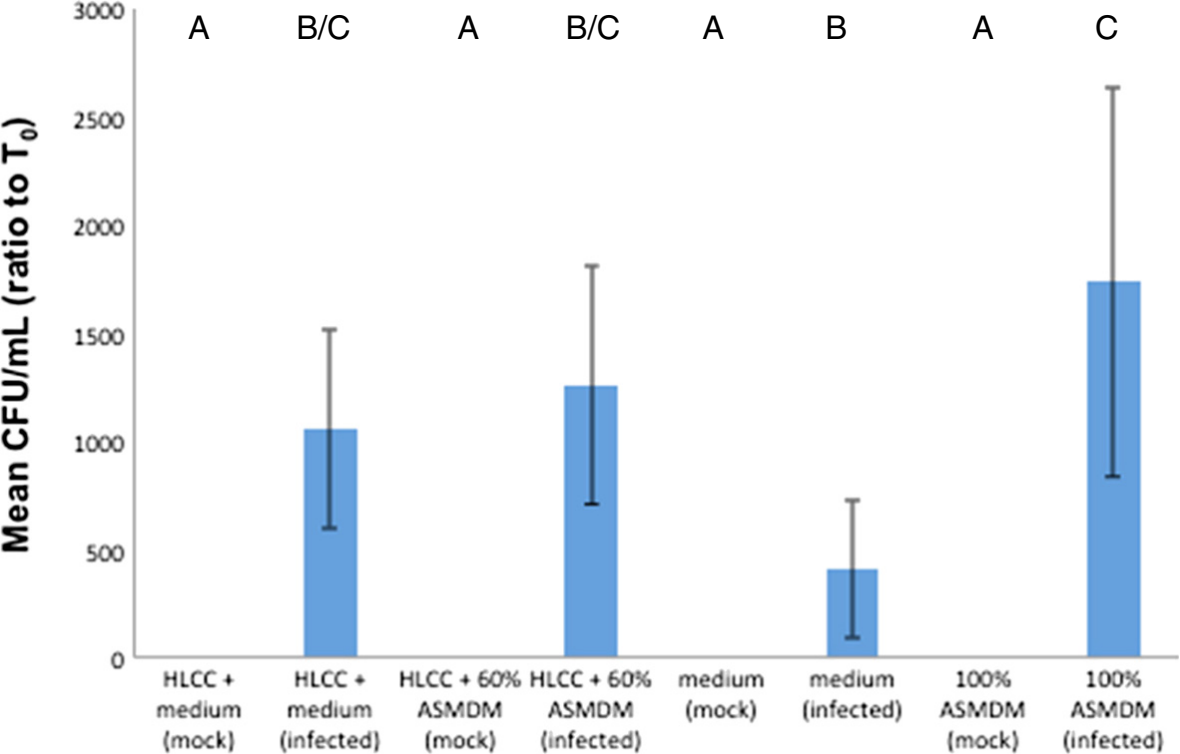
The effect of 60 % ASMDM and HLCC monolayers on *B. cenocepacia* growth. Confluent HLCCs or wells without HLCCs were exposed to 60 % ASMDM diluted in Ham’s F-12 complete medium, infected with *B. cenocepacia* at MOI 5 or mock-infected with saline, and incubated at 37 °C (7 % CO_2_). After 24 h, the bacterial density (CFU/mL) of each well was compared to the starting bacterial density (T_0_). There are statistically significant differences in mean CFU/mL ratio between the different treatments (ANOVA, *p* = 0.0003). A Tukey-HSD post-hoc test showed the mean CFU/mL ratio in infected 100 % ASMDM is significantly different than mock-infected HLCCs + medium, mock-infected HLCCs + 60 % ASMDM, infected or mock-infected medium, and mock-infected 100 % ASMDM (*p* = 0.0024 for all). The mean CFU/mL ratio in infected HLCCs + 60 % ASMDM is significantly different than mock-infected HLCCs + medium, mock-infected HLCCs + 60 % ASMDM, mock-infected medium, and mock-infected 100 % ASMDM (*p* = 0.0354). Values are averages of three independent experiments with duplicate wells. Bars show standard deviation. Variables with different letters are statistically different (oneway ANOVA, *p* < 0.05)

To assess the ability of *B. cenocepacia* to grow in the supernatant and inside the HLCC monolayer, the mean ratio of bacterial CFU/mL was determined. The bacterial levels were significantly high in the supernatants in all infected wells, and no bacterial growth was observed in any of the mock-infected wells (Fig. 7). A Tukey-HSD post-hoc test indicated that the infected 60 % ASMDM mean CFU/ml ratio is significantly higher than mock-infected HLCCs in medium, mock-infected HLCCs in 60 % ASMDM, mock-infected medium, and mock-infected 100 % ASMDM (Tukey-HSD post hoc, *p* = 0.0354 for all).

**Fig. 8:**
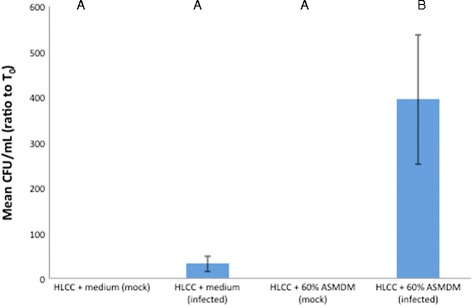
The effect of 60 % ASMDM on the ability of *B. cenocepacia* to invade the HLCC monolayer. Confluent HLCCs were exposed to 60 % ASMDM diluted in Ham’s F-12 complete medium, infected with *B. cenocepacia* at MOI 5 or mock-infected with saline, and incubated at 37 °C (7 % CO_2_). After 24 h, HLCCs were washed and then lysed with sterile dH_2_O. The bacterial density (CFU/mL) in each lysate was compared to the starting bacterial density at T_0_. Differences in mean ratio of CFU/mL were significantly different as indicated by oneway ANOVA (*p* = 0.0004). A Tukey-HSD post-hoc test indicated that the mean ratio CFU/mL in infected HLCCs + 60 % ASMDM is significantly different than in infected or mock-infected HLCCs + medium and in mock-infected HLCCs + 60 % ASMDM (*p* = 0.0007, *p* = 0.0007, and *p* = 0.0013, respectively). Values are averages of three independent experiments with single wells. Bars show standard deviation. Variables with different letters are statistically different (oneway ANOVA, *p* < 0.05)

Bacteria were recovered from the monolayer from all infected wells, but not from mock-infected wells (Fig. 8). The bacterial CFU/mL ratio in infected HLCCs grown in 60 % ASMDM was significantly greater than infected and mock-infected HLCCs in medium, and in mock-infected HLCCs in 60 % ASMDM (Tukey-HSD post hoc, *p* = 0.0007, *p* = 0.0007, and *p* = 0.0013, respectively) (Fig. 8). Collectively, *B. cenocepacia* grew well in the supernatant and associated with the HLCC layer.

## Discussion

Our goal was to develop an alternative, accessible model that mimics the environment of CF airways and PCD--another chronic airway disease--to investigate *B. cenocepacia* infections. In order to establish this model, we first needed to choose a cell line. CF epithelial cell lines are not economically feasible, so we used the human lung carcinoma A549 cell line (HLCCs). Next, we needed to show that HLCCs could survive in ASMDM. The ASMDM used in this study mimics the harsh conditions in CF sputum that bacterial pathogens would encounter; it is extremely viscous, acidic, and contains components detected in CF sputum [14]. Initially, we exposed HLCCs to 100 % ASMDM in regular tissue culture wells, but we noted a substantial drop in both cell viability and density (data not shown). We hypothesized the thick ASMDM prevented appropriate cellular gas exchange so we grew the HLCCs on gas permeable plates in 350 uL 100 % ASMDM. Again, viability and density were very low with a 100 % mortality. Instead, we tested a range of ASMDM diluted in Ham’s complete medium in order to find an appropriate balance of nutrients and gas exchange for the cells. We chose the 60 % ASMDM because it was the closest concentration that provided a healthy cell viability. Interestingly, there was a drop (although not statistically significant) in HLCC density in the presence of ASMDM, but this decrease was not detected by visual inspection because the center of the monolayers were intact. During the process of removing the ASMDM for subsequent washes, we noted that some of the periphery monolayer had detached. We think that the weight and thickness of the ASMDM caused the detachment. An additional image file shows this in more detail [see Additional file 2]. These results indicate that the cells survived ASMDM, but the ASMDM affected the robustness of the cell density.

Our second task was to show that *B. cenocepacia* could grow in the ASMDM. Not only did *B. cenocepacia* grow well, but the density increased over 100-fold in the presence of any ASMDM. The density increased the most (nearly 1000-fold) in the gas permeable plates which is understandable because these plates are designed to facilitate growth. These results align with the fact that *Pseudomonas aeruginosa*, another CF pathogen, is also able to grow well in this artificial sputum medium [14].

Finally, we wanted to create a model that shows *B. cenocepacia* damages the HLCCs. In the presence of 60 % ASMDM, *B. cenocepacia* associated with the HLCC monolayer in large numbers, compared to HLCCs in the presence of Ham’s F-12 complete medium alone. Similar results were obtained by Sajjan et al. [16], who found that *B. cenocepacia* was able to deeply invade the cell layer of infected CF airway tissue samples in the presence of a CF mucosal layer. However, *B. cenocepacia* largely remained on the surface of the mucosal layer in infected normal airway tissue samples in the presence of a normal mucosal layer [16]. The fact that *B. cenocepacia* associated with the cell layer in our model suggests that HLCC monolayers in 60 % ASMDM approximates the environment found in the CF airways, and that this model may be a good starting point to study *B. cenocepacia* infections in these airways.

Aside from the development of a new model to study *B. cenocepacia* infections in CF and PCD airways, the present study also indicates that CF sputum may play an important role in the pathogenesis of *B. cenocepacia*. Keeping in mind that ASMDM is similar to CF sputum, there are two pieces of evidence that support this idea. One, there was low viability of infected HLCCs in 60 % ASMDM compared to infected HLCCs in Ham’s F-12 complete medium. Two, there was a large number of bacterial cells found associated with the HLCC monolayers in 60 % ASMDM compared to HLCC monolayers in Ham’s F-12 complete medium. Collectively, these suggest that CF sputum, unlike normal sputum, may enable *B. cenocepacia* to reach and grow in or on the cell layers. In other words, unlike normal sputum, which serves as a protective layer against *B. cenocepacia* infection [16], CF sputum seems to enhance the pathogenesis of *B. cenocepacia*.

One possible explanation for this phenomenon is that a component of the ASMDM may activate *B. cenocepacia* motility by inducing the expression of genes such as the *rpoN* gene, which are required for motility [21], or flagellar structural genes. Drevinek et al. [22], for example, demonstrated that the expression of flagellar structural genes such as the *fliF* and *fliS* are upregulated in *B. cenocepacia* when grown in CF sputum, indicating that CF sputum properties may enhance *B. cenocepacia* motility [22]. CF sputum may also affect *B. cenocepacia* motility by enhancing its *cepRI* quorum sensing system, which controls certain motility genes [22, 23]. Moreover, previous studies suggest that some *B. cenocepacia* quorum sensing genes may be upregulated in CF sputum as well [22]. The signal that leads to increased *B. cenocepacia* motility in ASMDM may also originate from the eukaryotic host cells, rather than from the sputum. Eukaryotic host cells secrete certain signaling molecules in response to the inflammatory stress induced by initial *B. cenocepacia* infection or the presence of CF sputum. *B. cenocepacia* is known to elicit the secretion of inflammatory signals such as IL-8 by lung cells, for example [24, 25]. This signal may increase *B. cenocepacia* motility in a manner similar to the one described above, or it may even attract *B. cenocepacia* to the eukaryotic cell layer by means of chemotaxis. In this study, we found that the fold-increase in bacterial density is greater in infected wells containing HLCCs in either 60 % ASMDM or Ham’s F-12 complete medium than in infected wells void of cells, although these differences were not statistically significant. This supports the notion that the HLCCs could be releasing a signal detected by the bacteria.

Whether *B. cenocepacia* motility is increased randomly or directionally towards the eukaryotic host cells by way of chemotaxis, increased motility will likely lead to more bacteria being associated with the host cell layer. Once it reaches the host cell layer, *B. cenocepacia* could bind to and cause damage to eukaryotic cell layers, which could explain the decreased HLCC viability and degradation of the infected monolayer observed in 60 % ASMDM.

## Conclusion

The results of this study suggest that monolayers of HLCCs in 60 % ASMDM grown in gas-permeable plates serve as a viable starting point to investigate *B. cenocepacia* infections in patients with CF and PCD. This model might be used in the near future to further our understanding of this particular pathogen, and to develop new ways to eradicate this detrimental infection that, to this day, is still a significant cause of morbidity and mortality in CF patients.

## Additional files

Additional file 1: Bacterial clouds in F-12 medium and ASMDM wells. Confluent HLCCs were subjected to Ham’s F-12 complete medium (A and B) or 60 % ASMDM (C and D), infected with *B. cenocepacia* at MOI 0.3 (B and D) or mock-infected with saline (A and C), and incubated at 37 °C (7 % CO_2_). After 24 h, HLCC monolayers were assessed with an inverted Zeiss axiovert 40 CFL microscope (1000X magnification), a microscope-mounted camera, and Leica LAS v4.6.2. software. HLCC monolayers in Ham’s F-12 complete medium and 60 % ASMDM with *B. cenocepacia* show a dense mass in the upper left wells (arrows). Because this is seen in infected wells only, we think these are clouds of bacterial cells floating in the supernatant (B and D). Both mock-infected wells show no clouds (A and C). (DOCX 679 kb) Additional file 2: Detachment of cells with ASMDM exposure. HLCCs were added to gas-permeable plates at 4 × 10^5^ cells/mL. The plates were incubated at 37 °C (7 % CO_2_) for 24 h to form a monolayer. The medium was discarded, cells were washed once with 0.5 mL 1X HBSS, and then exposed to either 0.5 mL Ham’s F-12 complete medium or 60 % ASMDM suspended in Ham’s F-12 complete medium. Cells were mock-infected with saline, and incubated at 37 °C (7 % CO_2_). After 24 h, the monolayer was visualized at 400X magnification. The medium was discarded, cells were washed in 0.5 mL 1X HBSS, and visualized again. The F-12 periphery monolayer looks similar before (A) and after (B) the HBSS wash. In contrast, the 60 % ASMDM shows cell detachment (arrows) before (C) the HBSS wash, and there are larger, more noticeable gaps are seen after the wash (D). (DOCX 1977 kb)

## References

[1] Bush A, Payne D, Pike S, Jenkins G, Henke MO, Rubin BK (2006). Mucus properties in children with primary ciliary dyskinesia: comparison with cystic fibrosis. Chest.

[2] Noone PG, Leigh MW, Sannuti A, Minnix SL, Carson JL, Hazucha M, Zariwala MA, Knowles MR (2004). Primary ciliary dyskinesia: diagnostic and phenotypic features. Amer J Respir Crit Care Med.

[3] Afzelius BA (1976). A human syndrome caused by immotile cilia. Science.

[4] Mahenthiralingam E, Urban TA, Goldber JB (2005). The multifarious, multireplicon *Burkholderia cepacia* complex. Nat Rev Microbiol.

[5] LiPuma JJ (1998). *Burkholderia cepacia*: management issues and new insights. Clin Chest Med.

[6] Isles A, Maclusky I, Corey M, Gold R, Prober C, Fleming P, Levison H (1984). *Pseudomonas cepacia* infection in cystic fibrosis: an emerging problem. J Pediatr.

[7] Peeters C, Zlosnik JEA, Spilker T, Hird TJ, LiPuma JL, Vandamme P (2013). *Burkholderia pseudomultivorans* sp. nov., a novel *Burkhoderia cepacia* complex species from human respiratory samples and the rhizosphere. Sys Appl Microbiol.

[8] Quinn JP (1998). Clinical problems posed by multiresistant nonfermenting gram-negative pathogens. Clin Infect Dis.

[9] Loutet SA, Valvano MA (2010). A Decade of *Burkholderia cenocepacia* virulence determinant research. Infect Immun.

[10] McClean S, Callaghan M (2009). *Burkholderia cepacia complex*: epithelial cell-pathogen confrontations and potential for therapeutic intervention. J Med Microbiol.

[11] LiPuma JL, Dasen SE, Nielson DW, Stern RC, Stull TL (1990). Person-to-person transmission of *Pseudomonas cepacia* between patients with cystic fibrosis. Lancet.

[12] Baldwin A, Mahenthiralingam E, Drevinek P (2007). Environmental *Burkholderia cepacia* complex isolates from human infections. Emer Infect Dis.

[13] Govan JRW, Hughes JE, VanDamme P (1996). *Burkholderia cepacia*: medical, taxonomic and ecological issues. J Med Microbiol.

[14] Fung C, Naughton S, Turnbull L, Tingpej P, Rose B, Arthur J, Hu H, Harmer C, Harbour C, Hassett DJ, Whitchurch CB, Manos J (2010). Gene expression of *Pseudomonas aeruginosa* in a mucin-containing synthetic growth medium mimicking cystic fibrosis lung sputum. J Med Microbiol.

[15] Vonberg RP, Gastmeier P (2005). Isolation of infectious cystic fibrosis patients: Results of a systematic review. Infect Control Hosp Epidemiol.

[16] Sajjan U, Keshavjee S, Forstner J (2004). Responses of well-differentiated airway epithelial cell cultures from healthy donors and patients with cystic fibrosis to *Burkholderia cenocepacia* infection. Infect Immun.

[17] Darling P, Chan M, Cox AD (1998). Siderophore production by cystic fibrosis isolates of *Burkholderia cepacia*. Infect Immun.

[18] Tomlin KL, Clark SRD, Ceri H (2004). Green and red fluorescent protein vectors for use in biofilm studies of the intrinsically resistant *Burkholderia cepacia* complex. J Microbiol Methods.

[19] Burns JL, Jonas M, Chi EY, Clark DK, Berger A, Griffith A (1996). Invasion of respiratory epithelial cells by *Burkholderia* (*Pseudomonas*) *cepacia*. Infect Immun.

[20] Duff C, Murphy PG, Callaghan M, McClean S (2006). Differences in invasion and translocation of Burkholderia cepacia complex species in polarised lung epithelial cells in vitro. Microb Pathog.

[21] Saldias MS, Lamothe J, Wu R, Valvano MA (2008). *Burkholderia cenocepacia* requires the RpoN sigma factor for biofilm formation and intracellular trafficking within macrophages. Infect Immun.

[22] Drevinek P, Holden MTG, Ge Z, Jones AM, Ketchell I, Gill RT, Mahenthiralingam E (2008). Gene expression changes linked to antimicrobial resistance, oxidative stress, iron depletion and retained motility are observed when *Burkholderia cenocepacia* grows in cystic fibrosis sputum. BMC Infect Dis.

[23] Lewenza S, Visser MB, Sokol PA (2002). Interspecies communication between *Burkholderia cepacia* and *Pseudomonas aeruginosa*. Can J Microbiol.

[24] Reddi K, Phagoo SB, Anderson KD, Warburton D (2003). *Burkholderia cepacia*-induced IL-8 gene expression in an alveolar epithelial cell line: signaling through CD14 and mitogen-activated protein kinase. Pediatr Res.

[25] Palfreyman RW, Watson ML, Eden C, Smith AW (1997). Induction of biologically active interleukin-8 from lung epithelial cells by *Burkholderia* (*Pseudomonas*) *cepacia* products. Infect Immun.

