# Supplementary figures and images for: A novel method for investigating Burkholderia cenocepacia infections in patients with cystic fibrosis and other chronic diseases of the airways
Source: BMC Microbiol. 2016 Sep 1;16(1):200. doi: 10.1186/s12866-016-0811-7 (PMC5009706; doi:10.1186/s12866-016-0811-7)

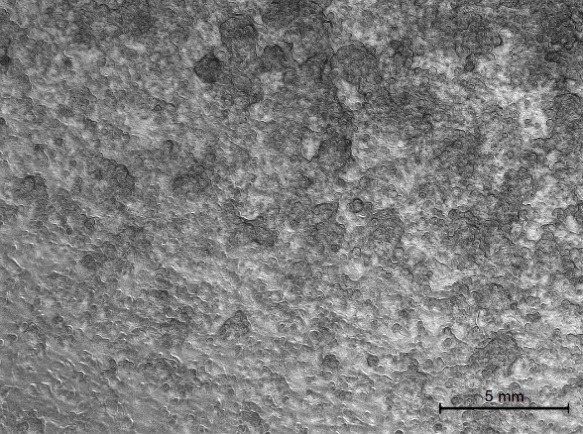

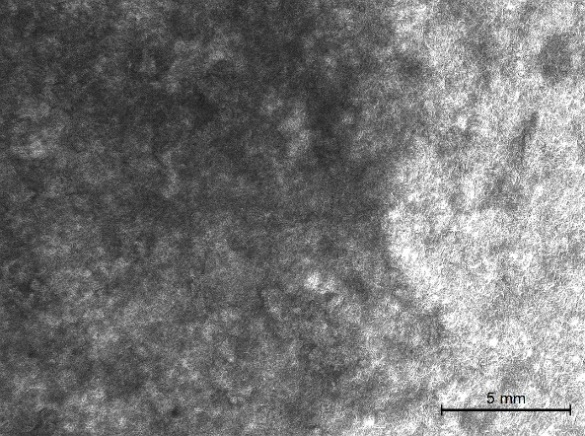


**B**

**A**

**C**


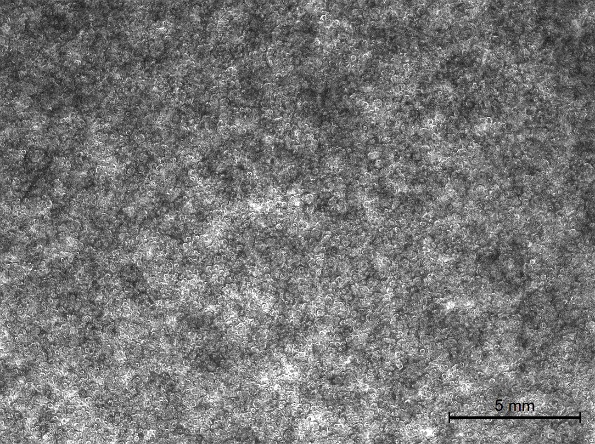

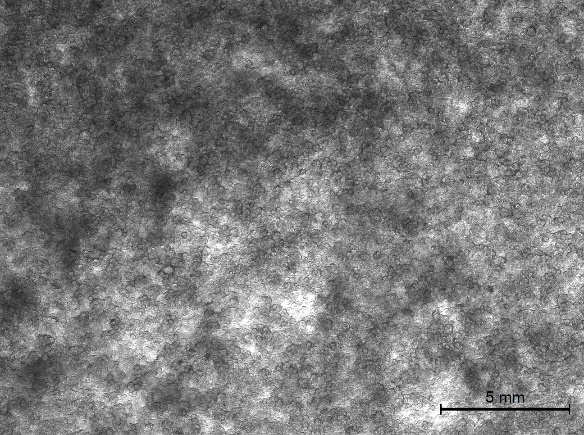


**D**

Supplement: Additional file 1: — Bacterial clouds in F-12 medium and ASMDM wells. Confluent HLCCs were subjected to Ham’s F-12 complete medium (A and B) or 60 % ASMDM (C and D), infected with B. cenocepacia at MOI 0.3 (B and D) or mock-infected with saline (A and C), and incubated at 37 °C (7 % CO2). After 24 h, HLCC monolayers were assessed with an inverted Zeiss axiovert 40 CFL microscope (1000X magnification), a microscope-mounted camera, and Leica LAS v4.6.2. software. HLCC monolayers in Ham’s F-12 complete medium and 60 % ASMDM with B. cenocepacia show a dense mass in the upper left wells (arrows). Because this is seen in infected wells only, we think these are clouds of bacterial cells floating in the supernatant (B and D). Both mock-infected wells show no clouds (A and C). (DOCX 679 kb) [file 12866_2016_811_MOESM1_ESM.docx]

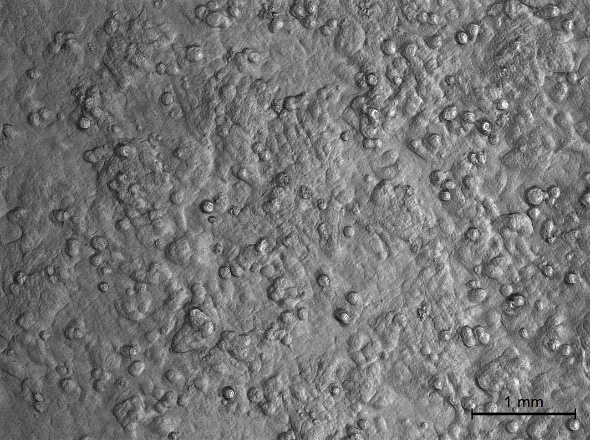

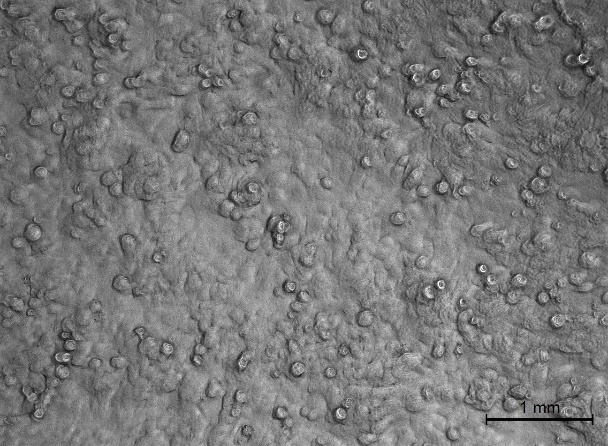

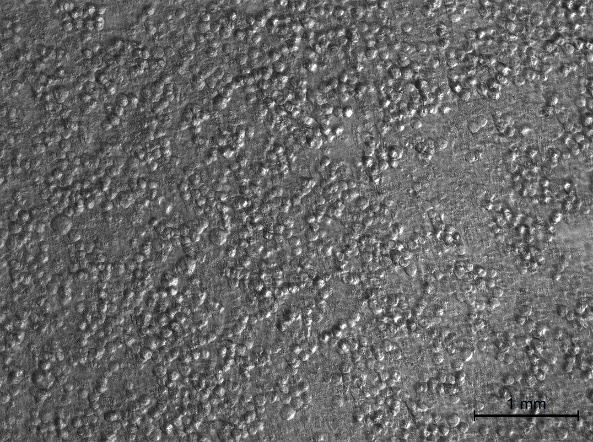

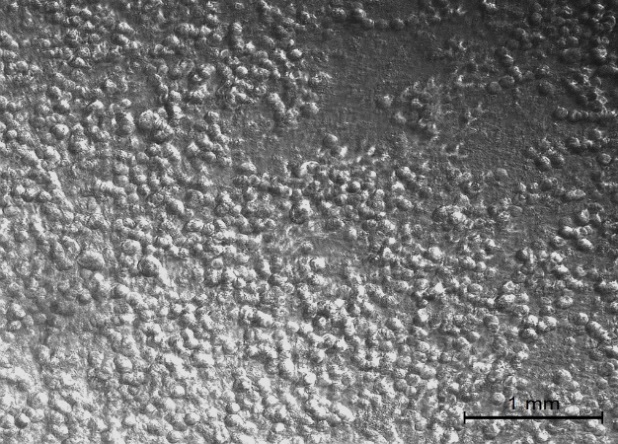


**A**

**B**

**C**

**D**

Supplement: Additional file 2: — Detachment of cells with ASMDM exposure. HLCCs were added to gas-permeable plates at 4 × 10^5^ cells/mL. The plates were incubated at 37 °C (7 % CO2) for 24 h to form a monolayer. The medium was discarded, cells were washed once with 0.5 mL 1X HBSS, and then exposed to either 0.5 mL Ham’s F-12 complete medium or 60 % ASMDM suspended in Ham’s F-12 complete medium. Cells were mock-infected with saline, and incubated at 37 °C (7 % CO2). After 24 h, the monolayer was visualized at 400X magnification. The medium was discarded, cells were washed in 0.5 mL 1X HBSS, and visualized again. The F-12 periphery monolayer looks similar before (A) and after (B) the HBSS wash. In contrast, the 60 % ASMDM shows cell detachment (arrows) before (C) the HBSS wash, and there are larger, more noticeable gaps are seen after the wash (D). (DOCX 1977 kb) [file 12866_2016_811_MOESM2_ESM.docx]
